# Platelet-rich plasma (PRP): an adjuvant to hasten hamstring muscle recovery. A randomized controlled trial protocol (ISCRTN66528592)

**DOI:** 10.1186/1471-2474-13-138

**Published:** 2012-08-06

**Authors:** Mohamad Shariff A Hamid, Mohamed Razif Mohamed Ali, Ashril Yusof, John George

**Affiliations:** 1 Sports Medicine Unit, Faculty of Medicine, University Malaya, Kuala Lumpur, 50603, Malaysia; 2 National Sports Institute of Malaysia, National Sports Complex, Bukit Jalil, 57000, Kuala Lumpur, Malaysia; 3 Sports Centre, University of Malaya, Kuala Lumpur, 50603, Malaysia; 4 University of Malaya Research Imaging Centre, Kuala Lumpur, 50603, Malaysia

## Abstract

**Background:**

Muscle injuries are one of the commonest injuries affecting athletes. It often leads to significant pain and disability causing loss of training and competition time. With current treatment, the duration to return-to-play ranges form six weeks to never, depending on injury severity. Recent researches have suggested that autologous platelet-rich plasma (PRP) injection into the injured site may hasten soft tissues healing. To-date, there has been no randomised clinical trials to evaluate the effects of PRP on muscle healing. The aim of this study is to examine the effects of autologous PRP on duration to return-to-play after muscle injury.

**Methods and design:**

A randomised, single blind controlled trial will be conducted. Twenty-eight patients aged 18 years and above with a recent grade-2 hamstring injury will be invited to take part. Participants will be randomised to receive either autologous PRP injection with rehabilitation programme, or rehabilitation programme only. Participants will be followed up at day three of study and then weekly for 16 weeks. At each follow up visit, participants will be assessed on readiness to return-to-play using a set of criteria. The primary end-point is when participants have fulfilled the return-to-play criteria or end of 16 weeks.

The main outcome measure of this study is the duration to return-to-play after injury.

**Conclusion:**

This study protocol proposes a rigorous and potential significant evaluation of PRP use for grade-2 hamstring injury. If proven effective such findings could be of great benefit for patients with similar injuries.

**Trial registration:**

Current Controlled Trials ISCRTN66528592

## Background

Muscle injuries are one of the commonest injuries affecting athletes [1]. They account for up to 30 – 50% of the injuries in sports events [2,3]. Majority of muscle injuries are results of excessive strain on muscle, which occurs during sprinting or jumping. Muscle injury may be the result of excessive eccentric contraction, when the muscle develops tension while lengthening [4]. This injury often affects the myotendinous junction of superficial muscles spanning across two joints, such as the rectus femoris, semitendinosus, and gastrocnemius muscles [1].

The diagnosis and grading of muscle injury is usually made through a thorough clinical assessment. Diagnostic ultrasound examination is often recommended as the method of choice for confirming and grading the muscle injury [5]. Despite the high frequency of muscle injury, the best method of its treatment has not yet been clearly defined. Currently, many interventions are used, guided by limited randomised controlled trials and quality prospective studies [6]. In professional sports, muscle injury often leads to significant pain and disability causing loss of training and competition time. Despite many treatment options, the duration of the return-to-play (RTP) period ranges from six weeks to never, based on the severity of the strains [7]. Current treatment includes rest, ice, compression and elevation (RICE) with a short period of immobilization during the early phase. In addition, short-term use of nonsteroidal anti-inflammatory (NSAIDs), corticosteroid medications and rehabilitation programmes is recommended [6,8-14].

Basic science of muscle healing has directed attention towards the use of autologous biological products as a treatment alternative for muscle injury. Damaged muscle goes through the early phase of destruction (inflammatory phase), where affected cells including muscles, blood vessels, connective tissues and intramuscular nerve undergo necrosis [15]. This phase is followed by repair and remodelling phases, in which undifferentiated satellite cells, in response to various growth factors, proliferate and differentiate into mature myoblasts in an effort to replace the injured muscle fibers [1]. Many of the growth factors are stored in the alpha (α) granules within platelets [16].

Inflammation occurring after muscle injury usually leads to accumulation of inflammatory cells, neutrophils and macrophages. Activation of platelets also occurs early at the injured site. Activated platelets degranulate releasing various substances, including growth factors. In addition, platelets contain other metabolically active substances such as adhesive proteins (TSP-1), clotting factors and their inhibitors (TFPI), proteases (MMP-1, 2 & 9 and TIMP1-4), chemokines (SDF-1α), cytokines and membrane glycoproteins (CD40L), involved in tissue repair and regeneration [16]. Platelet derived growth factors (PDGF), vascular endothelial growth factors (VEGF), epidermal growth factor (EGF), basic fibroblasts growth factors (bFGF), insulin-like growth factor-1 (IGF-1) and transforming growth factor beta-1 (TGF-β1) are some of the growth factors released by platelets [17]. IGF-1 and bFGF have the ability to accelerate healing following muscle and tendon injury [18]. A previous study from an animal model showed autologous PRP injection significantly hastens tibialis anterior muscle recovery from 21 days to 14 days [19]. Sanchez et al. at the 2nd World Congress on Regenerative Medicine 2005 presented a similar finding. They noted athletes receiving PRP injection under ultrasound guidance gain full recovery within half of the expected time [20]. In a study involving professional athletes, Wright-Carpenter et al. (2004) demonstrated autologous conditioned serum (ACS) injected into the injured muscle shortened the duration to full RTP by 30% (six days). They attributed the observed effects to the presence of increased levels of growth factors (FGF-2, HGF and TGF-β1) demonstrable on ELISA [7]. In 2010, the International Olympic Committee (IOC) concluded that currently there is very limited scientific evidence of clinical efficacy and safety profile of PRP use in athletic injuries [21]. More recently, a systematic review article, reported there has been no randomised clinical trials of PRP effects on muscle healing. In addition, only four clinical reports (level of evidence 3 or 4) were available [22]. More work on clinical science of PRP using robust clinical trials to demonstrate its efficacy has been recommended [21,22].

This paper describes the protocol of a randomised controlled trial to evaluate the clinical efficacy of a single injection of PRP combined with a rehabilitation programme on the duration to RTP after grade-2 hamstring injury. We hypothesized that distinct differences would be observed in the duration of RTP between those treated with combined PRP and rehabilitation programme versus rehabilitation programme alone. The presence of various growth factors in PRP could speed up muscle recovery.

## Methods and design

### Study design

This study involved a randomised, assessor-blinded controlled trial of 16-week duration. Participants were screened before enrolment. Measurements (described below) were taken upon study enrolment. On day three following the PRP injection, the participants were reviewed for any adverse reaction. Subsequently, all the participants were reassessed once a week until the end of the study period. The protocol conformed to the CONSORT guidelines for nonpharmacological interventions [23].

### Participants

Patients with confirmed grade-2 hamstring muscle injury were invited to participate in this study. Study notice and invitation to take part were distributed to all sports physicians practicing within Klang Valley, Selangor, Malaysia. The eligibility criteria for inclusion were as follows:

(i) Aged ≥ 18 years;

(ii) Acute hamstring muscle injury (≤ seven days);

(iii) Able to understand study protocol and completing the written informed consent.

The exclusion criteria were:

(i) Having received any form of injection therapy for current injury;

(ii) Using nonsteroidal anti-inflammatory drugs (NSAIDs) within one week before randomisation;

(iii) Unable to fulfil follow-up;

(iv) Significant cardiovascular, renal, hepatic disease, malignancy, history of anaemia, and previous muscle surgery.

### Procedure

**Figure 1:**
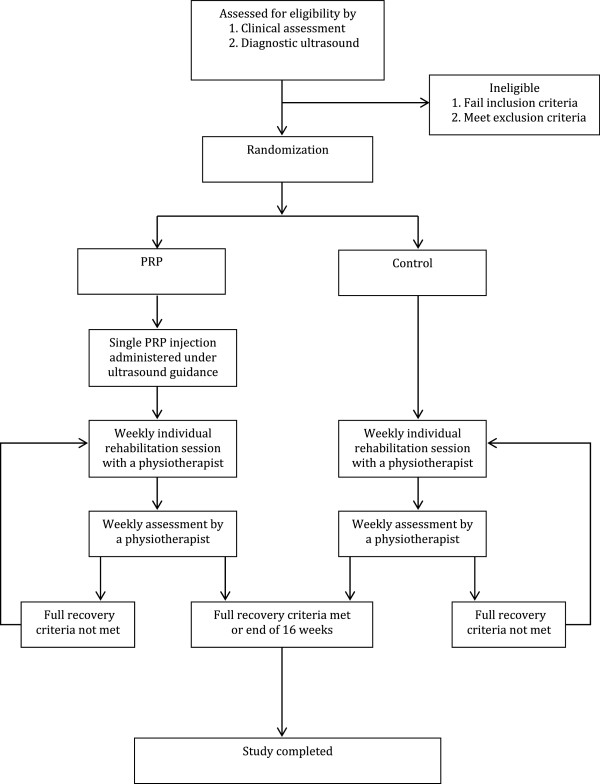
Summary of trial design.

The procedure is outlined in Figure 1. An initial screening was conducted at the Sports Medicine Clinic of the University of Malaya Medical Centre to determine injury severity. A sport physician and a physiotherapist conducted physical examination and grading of injury severity using clinical grading as recommended by Jarvinen et al. and DeLee et al. [24,25].

Later, two experienced musculoskeletal radiologists conducted a diagnostic ultrasound (Philips IU 22 ultrasound with 17–5 MHz Probe) to confirm the diagnosis, using the grading system used at our hospital (Table 1) and the grading suggested by Peetrons et al. [26]. Any disagreement between assessors was resolved through discussion. Diagnostic ultrasound assessment was conducted 24 to 48 hours after completion of physical examination. We also kept the record of those found to be ineligible. Patients with grade-2 hamstring muscle injury on clinical assessment and confirmed on diagnostic ultrasound examination were invited to participate.

**Table 1:** Grading of muscle strain injuries on ultrasound

**Grade**	**Ultrasound findings**
0	No ultrasound features seen
1	Muscle oedema only
2a	Partial tears of muscle fibres, disruption involving <33%
2b	Partial tears of muscle fibres, disruption involving ≥ 33 – 66%
2c	Partial tears of muscle fibres, disruption involving ≥ 66 - 99%
3	Complete tear of muscle

### Randomisation

Participants were randomly allocated into one of two groups: (i) autologous PRP group or (ii) control group. Randomisation was performed on those eligible after they had signed the written informed consent. A computer generated block randomisation of four was used to create a randomisation schedule. Treatment assignments were conducted by the trial manager MS.

### Blinding

Three physiotherapists (PC, FJ, SR) acted as the outcome measure assessors. They were involved in providing standard rehabilitation programmes to all participants but were blinded about the participant group allocation. The participants were requested not to disclose details of their treatment. On trial completion, the assessor was asked to guess which treatment each participant received. The success of blinding was determined by calculating the ‘blinding index’ using the method demonstrated by James et al. [27].

### Interventions

#### PRP intervention group

Participants in the PRP group received a single injection of autologous PRP under expert ultrasound guidance by a musculoskeletal radiologists trained in interventional musculoskeletal injections. The injection was administered once, following randomisation of the treatment group (day 1 of the study).

#### PRP preparation

Fifty-five millilitres (ml) of venous blood were collected from the participants’ arm into a 60 ml syringe primed with ACD-A. In addition, 2 ml of venous blood were collected and sent to the hospital laboratory for determination of platelets and leucocytes count. The blood collected for PRP was prepared according to the GPS^TM^ III Systems instruction for use (Biomet Biologics, Inc., Warsaw, Ind). Since an acidic anticoagulant was added during the collection of whole blood, PRP was buffered to increase the pH to normal physiological levels. This was accomplished by adding 8.4% sodium bicarbonate solution in a ratio 0.05 ml of sodium bicarbonate to 1 ml of PRP. No activating agent was added to the PRP. The time taken to prepare PRP was about 30 minutes. A standard 60 ml GPS^TM^ III kit could produce approximately 6 ml of PRP.

In our study, 3 ml of extracted PRP were injected into the injured area under ultrasound guidance. One ml was sent to the hospital laboratory for platelets and leucocyte count, while the remaining 2 ml were stored in −20° Celsius for analysis of growth factors (basic fibroblast growth factor [bFGF]; insulin-like growth factor-1 [IGF-1]; transforming growth factor-β1 [TGF-β1]), which were done later.

### Injection technique

As a recent study showed, a statistically significant decrease in tenocyte proliferation and cell viability, following PRP combined with the local anaesthetic agent (lidocaine and bupivacaine) [28], no local anaesthetic was given prior to PRP injection in the current study.

To the best of our knowledge the current existing guideline lacks information on the optimal timing, frequency of administration, clinical effective dose and volume, as well as post-injection rehabilitation technique following PRP injection for muscle injury [21,29]. Furthermore, no long-term clinical studies exist on potential adverse effects. Our decision to use a single injection of 3 ml of PRP in the intervention group was based on the findings of existing clinical studies. Sanchez at al. reported ultrasound guided injection of autologous preparation rich in growth factors (PRGF) within the injured muscle enhances healing and functional recovery. Further, small tears indicated good progress with a single application of PRGF, while a medium to large size tears required two or three applications of PGRF [20]. Hamilton et al. reported single injection of PRP combined with daily physiotherapy programme was effective for grade II semimembranosus strain injury. They demonstrated 17 days following injection of 3 ml PRP, the athlete was pain free and able to achieve full range of motion. The athletes were back to their preinjury activities after 3 weeks [30].

Under ultrasound guidance, 3 ml of PRP were injected directly into the injured area via an 18 G needle using a peppering technique. All injections were done under aseptic technique. Each participant in the PRP combined rehabilitation programme group received a single injection of PRP throughout the study. Immediately after injection, the patient was kept in supine position for 10 to 15 minutes. Participants were advised to rest, limit their activities for the next 48 hours, and use only acetaminophen for pain. The use of non-steroidal medication was prohibited.

Participants were reassessed for any adverse reaction three days after receiving PRP. Later, weekly reassessment was conducted until the end of the study. All participants were asked to continue with an unsupervised daily home exercise programmes as prescribed and to keep a record of these sessions. The use of painkillers, other than nonsteroidal anti-inflammatory drugs, was allowed. All medication use was recorded.

Participants in both groups were required to attend a weekly rehabilitation session with a physiotherapist until full recovery or the end of 16 weeks. At each visit, outcome measures were assessed, and rehabilitation programmes were conducted under a physiotherapist’s supervision. Each treatment session lasted for 45 – 60 minutes. Three-experienced physiotherapists (with at least five years of clinical experience) practicing at University Malaya Medical Centre and National Institute of Sports were trained to assess outcome measures and deliver rehabilitation programmes. The training involved a half-day course delivered by the principal researcher and a treatment manual. The treatment manual contained a brief summary of the study, assessment methods and hamstring rehabilitation based on the programme used by Sherry et al. [31]. In addition, the participants were expected to independently track their exercise compliance by recording the days they performed the prescribed rehabilitation programme on a logbook and to report any difficulties at each follow-up visit. The rehabilitation programme used in the study focused on progressive agility and trunk stabilization exercises (Table 2). This programme was based on a set of exercises used in an earlier study [31]. Further, this programme was found to be more effective than a programme that only emphasized on hamstring stretching and strengthening in promoting RTP and preventing injury recurrence in athletes affected with an acute hamstring strain [31].

**Table 2:** Rehabilitation programme

**Phase 1**	
1.	Low to moderate-intensity sidestepping, 3 × 1 min
2.	Low to moderate-intensity grapevine stepping (lateral stepping with the trail leg going over the lead leg and then under the leg), both directions, 3 × 1 min
3.	Low to moderate-intensity steps forward and backward over a tape line while moving sideways, 2 × 1 min
4.	Single-leg stand progressing from eyes open to eyes closed 4 × 20 sec
5.	Prone abdominal body bridge (performed by using abdominal and hip muscle to hold the body face-down straight-plank position with the elbows and feet as the only point of contact), 4 × 20 sec
6.	Supine extension bridge (performed by using abdominal and hip muscles to hold the body in a supine hook lying position with the head, upper back, arms, and feet as the points of contact), 4 × 20 sec
7.	Side bridge, 4 × 20 sec on each side
8.	Ice in long-sitting position for 20 min
Phase 2*	
1.	Moderate to high-intensity sidestepping, 3 × 1 min
2.	Moderate to high-intensity grapevine stepping, 3 × 1 min
3.	Moderate to high-intensity steps forward and backward while moving sideways, 2 × 1 min
4.	Single-leg stand windmill touches, 4 × 20 sec of repetitive alternate hand touches
5.	Push-up stabilization with trunk rotation (performed by starting at the top of a full push-up, then maintain this position with 1 hand while rotating the chest toward the side of the hand that is being lifted to point toward the ceiling, pause and return to the starting position), 2 × 15 reps on each side
6.	Fast feet in place (performed by jogging in place with increasing velocity, picking the foot only a few inches off the ground), 4 × 20 sec
7.	Proprioceptive neuromuscular facilitation trunk pull-downs with Thera-Band, 2 × 15 to the right and left
8.	Symptom-free practice without high-speed manoeuvres
9.	Ice for 20 min if any symptoms of local fatigue or discomfort present

Key: Low intensity, a velocity of movement that is less than or near that of normal walking; moderate intensity, a velocity of movement greater than normal walking but not as great as sport; high intensity, a velocity of movement similar to sport activity.

* Participants allowed to progress from phase 1 to phase 2 when they could walk with a normal gait pattern and perform a high knee march in place without pain.

### Primary outcome measures

In this study, primary outcome was the duration of RTP. Duration of RTP is defined as the duration (days) from the date of injury until the participants fulfil the criteria for RTP. The decision on determination of fitness for RTP is based on expert opinion [32]. As there were limited scientific studies done to examine the outcome of various RTP strategies [33], we decided to come up with our own criteria of RTP (Table 3) based on recent clinical sports medicine recommendations [8,34-37].

**Table 3:** Criteria for return-to-play (RTP)

**Sign**	**General Recommendation**
Pain	Pain-free (on direct palpation)
	Pain free on hamstring contraction (resisted isometric hamstring muscle contraction)
Range of motion	Symmetrical with unaffected site
Strength	Isokinetic strength within 5% [29,30] to 10% [8] of contralateral side

Direct hamstring palpation was conducted and pain elicited was recorded in the participants’ clinical research form (CRF). Pain provocation test was evaluated by isometric contraction of the hamstring muscles when palpation did not elicit any tenderness. This test was performed in prone lying with the knee flexed at approximately 15° [38]. Hamstring range of movement (ROM) was assessed using the active knee extension (AKE) test. The AKE test involves movement of the knee joint but not the hip, unlike the straight-leg raise (SLR) test which involves movements of both hip and knee joints. AKE test is an active test and is considered safe as the participants dictate the end point. This test has been recommended and often used to measure hamstring tightness. AKE test normal values of knee motion were reported to be within 20° on full extension of the knee [39].

Hamstring muscle strength was assessed using an isokinetic dynamometer (System 4 Pro, Biodex Medical System, NY, USA). Assessment of hamstring and quadriceps muscles of both legs was also conducted during participants’ weekly visit. Participants were allowed to familiarise with the experimental protocol before testing. During the familiarization period, participants practiced with sub-maximal effort. The participants’ knee joint centre was kept aligned with the axis of the dynamometer crank arm. The testing protocol included maximum voluntary strength of both legs, with the uninjured leg tested first. Muscle strength test was performed under concentric exertion at three angular speeds (60°, 180° and 240°/second). Each participant performed five maximum contractions at angular speeds of 60°/s, ten maximum contractions at angular speeds of 180°/s, and fifteen maximal contractions at angular speeds of 240°/s, with a rest interval of about 60 seconds between each speed. At each speed, quadriceps muscles were tested first followed by the hamstrings. The participants did not receive any visual feedback during the test; however, verbal encouragements were given.

The participants that failed to meet the RTP criteria at the end of week 16 were allowed to continue their treatment in the UMMC until full recovery.

### Secondary outcome measures

The Brief Pain Inventory - Short Form (BPI-SF) questionnaire were used to assess the severity and impact of pain on the participants’ daily functions. The BPI-SF is a self-reported questionnaire. It consists of four questions related to pain severity and seven questions related to pain interference on daily functions. The pain intensity items are presented as numeric rating scales, with a minimum score of 0 (indicating no pain) and a maximum score of 10 (when pain is as bad as one could imagine). Similar scales are used for the seven items on interference of participants’ daily functions. The BPI-SF has been validated in several languages, including Malay [40] and demonstrated a Cronbach alpha reliability that ranges from 0.77 to 0.91 [41].

Platelet levels in participants’ venous blood and PRP were determined. In addition, levels of insulin-like growth factor-1 (IGF-1), basic fibroblasts growth factor (bFGF) and transforming growth factor-beta 1 (TGF-β1) were determined using ELISA kits (Cusabio, USA).

The participants’ attendances to the physiotherapy session were recorded to determine adherence. In addition, their daily logbook of self-home exercise was also evaluated.

Any adverse events occurring during the study were documented and proper measures were taken.

### Sample size

Sample size was determined using the following formula [42]:

(1) $$N=\frac{2\times {\left[z_{\left(1-\alpha /2\right)}+z_{\left(1-\beta \right)}\right]}^{2}\sigma^{2}}{{\left[\mu_{1}-\mu_{2}\right]}^{2}}$$

Where:

*N* = the sample size in each of the groups

$z_{\left(1-\alpha /2\right)}\;\text{of }.05$= 1.96 (percentage of the normal distribution for statistical significance level of .05)

$z_{\left(1-\beta \right)}\;\text{of }80\%$= .84 (percentage of the normal distribution for statistical power of 80%)

μ_1_ = population mean in treatment Group 1

μ_2_ = population mean in treatment Group 2

μ_1_ – μ_2_ = the mean difference

σ^2^ = population [standard deviation (SD)]

Total sample size after estimation of 30% attrition rate

= 11 + 3 = 14 participants in each intervention group giving a total of 28 participants altogether [7].

### Data and statistical analysis

The primary analysis was done using the principle of intention-to-treat (ITT). ITT analysis includes participants with incomplete data, those who deviated from the study protocol and those who withdrew from the study. Missing data were handled through multiple imputation methods [43].

Socio-demographic, clinical characteristics and baseline information were presented to assess comparability between groups. Similar variables were also examined among the participants who withdrew from the study.

The primary endpoint of the study was the date when RTP was achieved or the end of week 16. Differences for categorical variables are tested with a chi-square test or Fischer’s exact test. As clinical outcome variables were repeatedly measured over time, a multivariate analysis of variance (MANOVA) for repeated measures was performed to explore an overall time, general group, and the time by group interaction effect.

Signs and symptoms changes were explored using linear regression analysis to determine the rate of change. In addition, levels of the various growth factors (IGF-1, bFGF and TGF) were determined. Statistical analyses were carried out using SPSS (Version 19). For all analyses, a value of *P* <.05 was considered significant.

### Timelines

The study was approved by the University Malaya Medical Centre (UMMC), Medical Ethics Committee in February 2011 (MEC Ref. No: 835.11). Recruitment and training of physiotherapists were conducted in September 2011. Patient recruitment started from February 2012. Expected completion date of the study is in December 2012.

## Discussion

This is the first randomised controls study to examine the effect of PRP on duration of RTP after a grade-2 hamstrings injury. There are several major strengths of the intervention design in this study. The primary outcome of this study includes a combination of subjective and objective assessments of RTP criteria. The criteria used are based on several current recommendations from leading experts and reflect present clinical practice [8,34-37].

The rehabilitation programme for this study has been based on a contemporary programme that was effective for acute hamstring strain. The average (±SD) time needed to RTP in athletes under such a rehabilitation programme was 37.4 ± 27.6 days [31].

Grade-2 muscle injury is confirmed on ultrasound (US) assessment. US is a cheap, reproducible and well-tolerated imaging examination, which also provides a real-time functional assessment in multiplanar views [44]. US is suggested to have equal sensitivity to MRI for acute hamstring muscle complex injury, especially when performed within 2 weeks following injury [45]. US assessment of hamstring injury in our study would ensure uniformity of injury grading and allow comparison of treatment interventions between groups.

Infiltration of autologous PRP under ultrasound guidance allows accurate delivery of PRP contents to the site where it is to have the greatest effect [46].Finally, levels of growth factors including IGF-1, bFGF and TGF-β1 in the PRP are determined using ELISA kits. This would allow us to explore the potential individual effect of PRP constituents on muscle healing.

## Conclusion

This is a randomised controlled trial exploring the effectiveness of a single injection of autologous PRP combined with hamstring rehabilitation programme on the duration of RTP after a grade-2 hamstring injury. The major strengths of this study include reproducibility and reflection of current clinical management of grade-2 hamstring injury. The findings enable recommendations of this treatment alternative for grade-2 hamstring injury.

## Competing interest

None of the authors has any competing interest arising from this research.

## Authors’ contributions

MSAH, MRMA, AY and GJ were responsible for identifying the research question, and contributing to drafting of the study protocol. All authors provided comments on the drafts and have read and approved the final version.

## Pre-publication history

The pre-publication history for this paper can be accessed here:

http://www.biomedcentral.com/1471-2474/13/138/prepub
